# Imaging gas in a com­bus­tion engine with high-energy X-ray Compton scattering

**DOI:** 10.1107/S1600577526002031

**Published:** 2026-04-02

**Authors:** Yuki Mizuno, Hiroaki Suzuki, Naruki Tsuji, Takuyo Oguchi, Go Matsubara, Hiroyuki Yamase, Yoshiharu Sakurai

**Affiliations:** a Japan Synchrotron Radiation Research Institute Sayo Hyôgo679-5198 Japan; b Japan AeroSpace Technology Foundation (JAST), Sendai, Miyagi980-0811, Japan; c Research Center of Materials Nanoarchitectonics (MANA) National Institute for Materials Science Tsukuba Ibaraki305-0047 Japan; NSRRC, Taiwan

**Keywords:** X-ray Compton scattering, internal com­bus­tion engine, time-resolved technique, gas temperature

## Abstract

We have developed a time-resolved Compton-scattering imaging technique for visualizing gas behaviours in operating internal com­bus­tion engines. This technique employs a digital processing system to record both the pulse height and the timing of detected X-rays, enabling the derivation of Compton-scattered X-ray spectra for each crank angle, which corresponds to the piston position. The observed crank-angle dependency of Compton-scattered X-ray intensity allowed us to successfully capture the density changes of the gas in the com­bus­tion chamber associated with piston motion and com­bus­tion. In addition, the capability of temperature estimation is discussed with a combination of X-ray Compton-scattering experiments and pressure measurements. This study demonstrates that time-resolved Compton-scattering imaging is a powerful and unique technique for characterizing gas behaviours in an operating internal com­bus­tion engine.

## Abstract 2

Compton-scattering imaging has been applied to a com­bus­tion engine under operation for the first time. With a time-resolved technique, a crank-angle dependency of Compton-scattered X-ray intensity is obtained at different positions in a com­bus­tion chamber.

## 1. Introduction

For a more sustainable global environment, improving fuel efficiency and reducing exhaust gas emissions in internal com­bus­tion engines are essential. The com­bus­tion process in an engine cylin­der is highly complex, governed by the dynamic distribution of fuel gas, combusted gas, temperature, ignition points and other factors. To better understand these intricate phenomena and enhance engine performance, direct in-cylin­der visualization has been pursued using optically accessible engines (Bowditch, 1961 ▸; Zhao, 2012 ▸). These engines are equipped with optical windows that allow visible laser beams to be directed into the cylin­der. However, the additional equipment and modifications required for optical access may influence the in-cylin­der com­bus­tion process.

To overcome this limitation, we have been developing a time-resolved Compton-scattering imaging (CSI) technique for direct observation of in-cylin­der gas behaviour. A key advantage of CSI over optical laser-based techniques is that it does not require any modifications to the engine. This is because CSI uses high-energy X-rays with high material transmittance, allowing penetration into the engine. Furthermore, even at high X-ray energies, Compton scattering exhibits a sufficient cross-section for light elements, making it suitable for visualizing in-cylin­der gas behaviours.

Besides internal com­bus­tion engines, CSI has been applied as a visualization technique for investigating the behaviour of light-element materials inside palm-size energy devices. Using high-energy X-rays with high material transmittance, CSI has been used successfully to observe dynamic phenomena in lithium-ion batteries and polymer electrolyte fuel cells. For example, migration of lithium in a commercial battery (Itou *et al.*, 2015 ▸; Suzuki *et al.*, 2020 ▸; Suzuki *et al.*, 2021 ▸) and the formation of liquid water in a fuel cell during operation (Tsuji *et al.*, 2021 ▸; Miyazawa *et al.*, 2023 ▸) have been clearly visualized using this method.

In a previous work on com­bus­tion gases, a self-sustaining flame made by a cylindrical Bunsen burner was measured using a scanning-type CSI technique (Sakurai *et al.*, 2016 ▸; Sakurai *et al.*, 2021 ▸). This result shows a large change in the intensity of Compton-scattered X-rays across the flame, due to variations in gas temperature. The intensity of Compton-scattered X-rays, d*N*, is given by
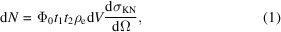
where Φ_0_ is the incident photon flux to the engine, *t*_1_ is the incident X-ray transmittance to the probing volume, *t*_2_ is the scattered X-ray transmittance from the probing volume to the X-ray detector, ρ_e_ is the average electron density over the probing volume d*V* and 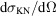 is the Klein–Nishina differential cross-section (Sharaf, 2001 ▸). Since *t*_1_ and *t*_2_ are assumed to be constant, the intensity of Compton-scattered X-rays, d*N*, probes the average electron density ρ_e_. The ratio of molecular density to electron density, α, is defined by
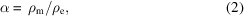
where ρ_m_ is the average molecular density. For the ideal gas, the temperature *T* is related to ρ_e_ by
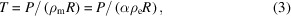
where *P* is the pressure and *R* is the ideal gas constant. The self-sustaining flame system can be treated under the ideal gas law (Tieng *et al.*, 1992 ▸). It should be noted that the thermodynamic cycle of internal com­bus­tion engines is described by an Otto cycle, which is based on the ideal gas model.

In this study, we applied the CSI technique to an operating internal com­bus­tion engine and visualized the crank-angle dependency of Compton-scattered X-ray intensity at various positions inside the com­bus­tion chamber. We also attempted to estimate the crank-angle dependency of the gas temperature in the chamber.

## 2. Experiment

**Figure 1:**
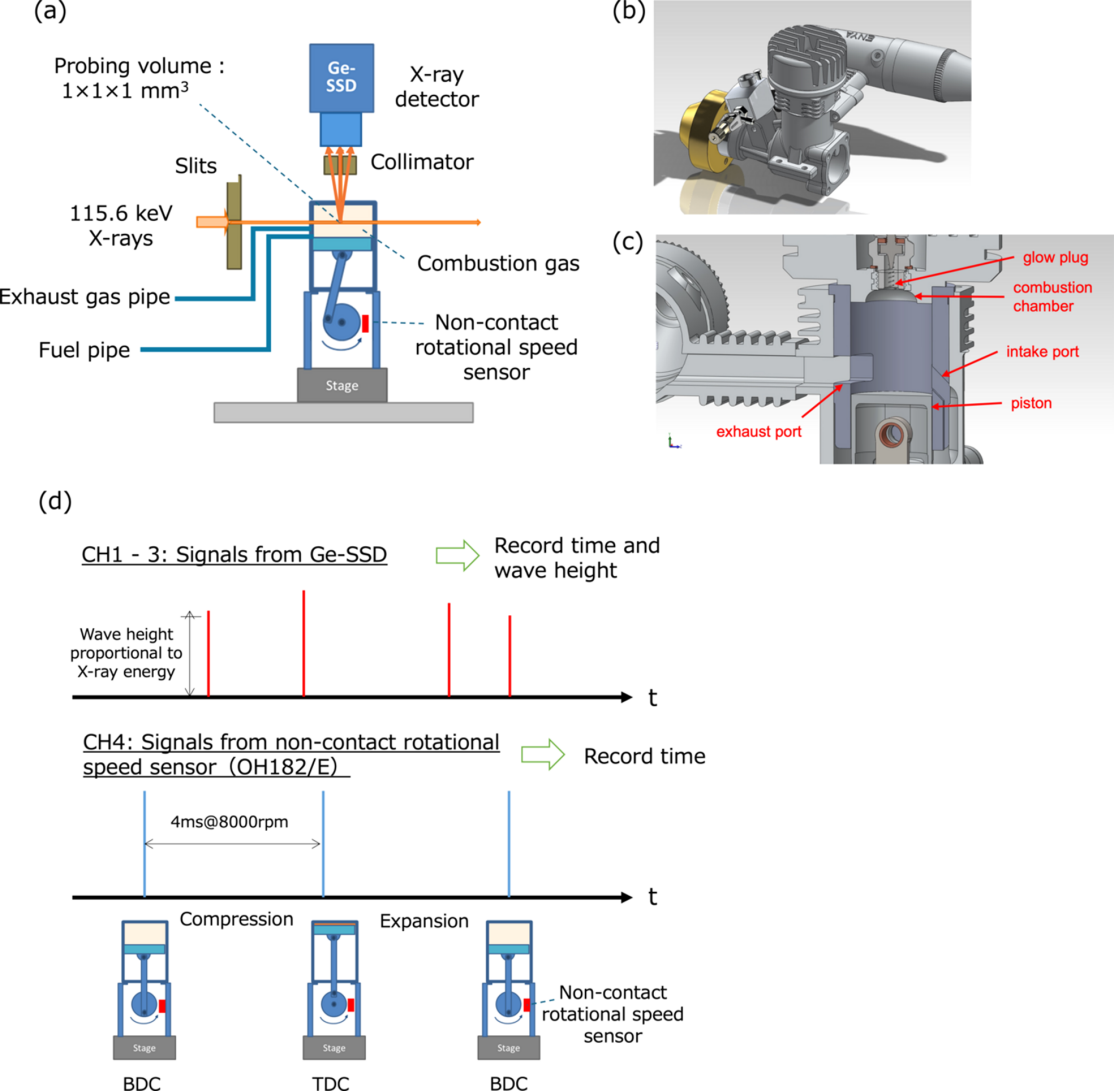
Schematic diagrams of the engine and experimental setup. (*a*) Schematic diagram of the experimental setup, (*b*) 3D drawing of the compact engine, (*c*) schematic diagram of the interior of the engine when the piston is at bottom dead centre (BDC) and (*d*) conceptual diagram of the time-resolved measurement.

The time-resolved Compton-scattering imaging was per­formed at the High Energy Inelastic Scattering (BL08W) beamline (Sakurai, 1998 ▸) at SPring-8. The X-ray source is an elliptical multipole wiggler operated in the linear polarization mode (Maréchal *et al.*, 1998 ▸). The synchrotron radiation X-rays are monochromated and focused horizontally by an asymmetric Johann-type Si monochromator with (400) reflecting planes (Yamaoka *et al.*, 2000 ▸) to deliver 115.6 keV X-ray beams. A schematic of the experimental setup is illustrated in Fig. 1 ▸(*a*). The entrance slit system defined the size of the incident X-rays to 1 mm in both height and width. X-rays scattered from the gas inside the com­bus­tion chamber passed through a collimator with nine square apertures (1 mm × 1 mm each) arranged in a 3 × 3 array and were detected by the nine elements of a Ge solid-state detector (Ge-SSD). Therefore, the probing volume was approximately 1 mm^3^. The intensity of the incident X-ray beams was monitored by an ion chamber for data normalization. The incident X-ray flux is estimated to be approximately 3 × 10^12^ photons s^−1^.

A three-dimensional drawing of the compact model engine is shown in Fig. 1 ▸(*b*). The engine used in this study is a two-stroke engine with a displacement of 3.5 cc, manufactured by Enya Metal Products Co. Ltd. It has a cylin­der bore diameter of 16.6 mm and a piston stroke length of 16.0 mm, resulting in a bore-to-stroke ratio close to 1. The com­bus­tion chamber volume is 306.79 mm^3^, while the total cylin­der volume is 3462.79 mm^3^. In addition, since the volume when the exhaust port is closed is approximately 2400 mm^3^, the com­pres­sion ratio is about 8. The wall of the com­bus­tion chamber is made of an aluminium–silicon alloy with a thickness of 5 mm, and the X-ray transmittance at 115 keV is approximately 80%. This high transmittance ensures that the experiment can be per­formed without significant attenuation effects. The engine operates on a fuel mixture consisting of 90% methanol and 10% lubricating oil. The air–fuel mixture is formed at a stoichiometric air–fuel ratio of 6.4 or slightly lower and introduced into the engine. Fig. 1 ▸(*c*) shows a schematic diagram of the interior of the engine when the piston is at the bottom dead centre (BDC). The glow plug is heated to assist ignition only when the engine is started. At BDC, the intake port is open, allowing the air–fuel mixture to enter the com­bus­tion chamber. As the piston moves upward, the intake and exhaust ports close, and the mixture is compressed. At top dead centre (TDC), the compressed mixture ignites and burns, generating com­bus­tion pressure that drives the piston downward. As the piston descends, the exhaust port opens and the com­bus­tion gases are expelled. Further piston descent reopens the intake port, drawing in a fresh air–fuel mixture while expelling residual com­bus­tion gases through the exhaust port, completing the cycle. The engine operates through continuous repetition of these two processes – intake/com­pres­sion and com­bus­tion/exhaust – driven by the reciprocating motion of the piston. In this experiment, the engine was operated at approximately 8000 to 9000 rpm. Measurements were conducted for one hour at each of seven locations in the horizontal direction inside the com­bus­tion chamber.

Fig. 1 ▸(*d*) shows a conceptual diagram of the time-resolved measurement. Signals from three Ge-SSD elements were recorded using TechnoAP’s Digital Signal Processor (DSP) APN504X at channels CH1, CH2 and CH3, along with their respective acquisition times. Due to the limited number of DSP channels, only three of the nine elements of the Ge solid-state detector positioned directly above the incident X-ray beam were used. Additionally, signals from a non-contact rotational speed sensor (OH182/E), which generates signals when the piston reaches TDC and BDC, were recorded at CH4, along with their acquisition times. Based on the data acquisition time and the times at which the piston reaches TDC and BDC, it is possible to determine the piston position at the moment of data acquisition. The travel time from BDC to TDC and that from TDC to BDC are each divided into 20 intervals, and signals within each time interval are accumulated, yielding spectra corresponding to 40 discretized piston positions (crank angles).

## 3. Results and discussion

**Figure 2:**
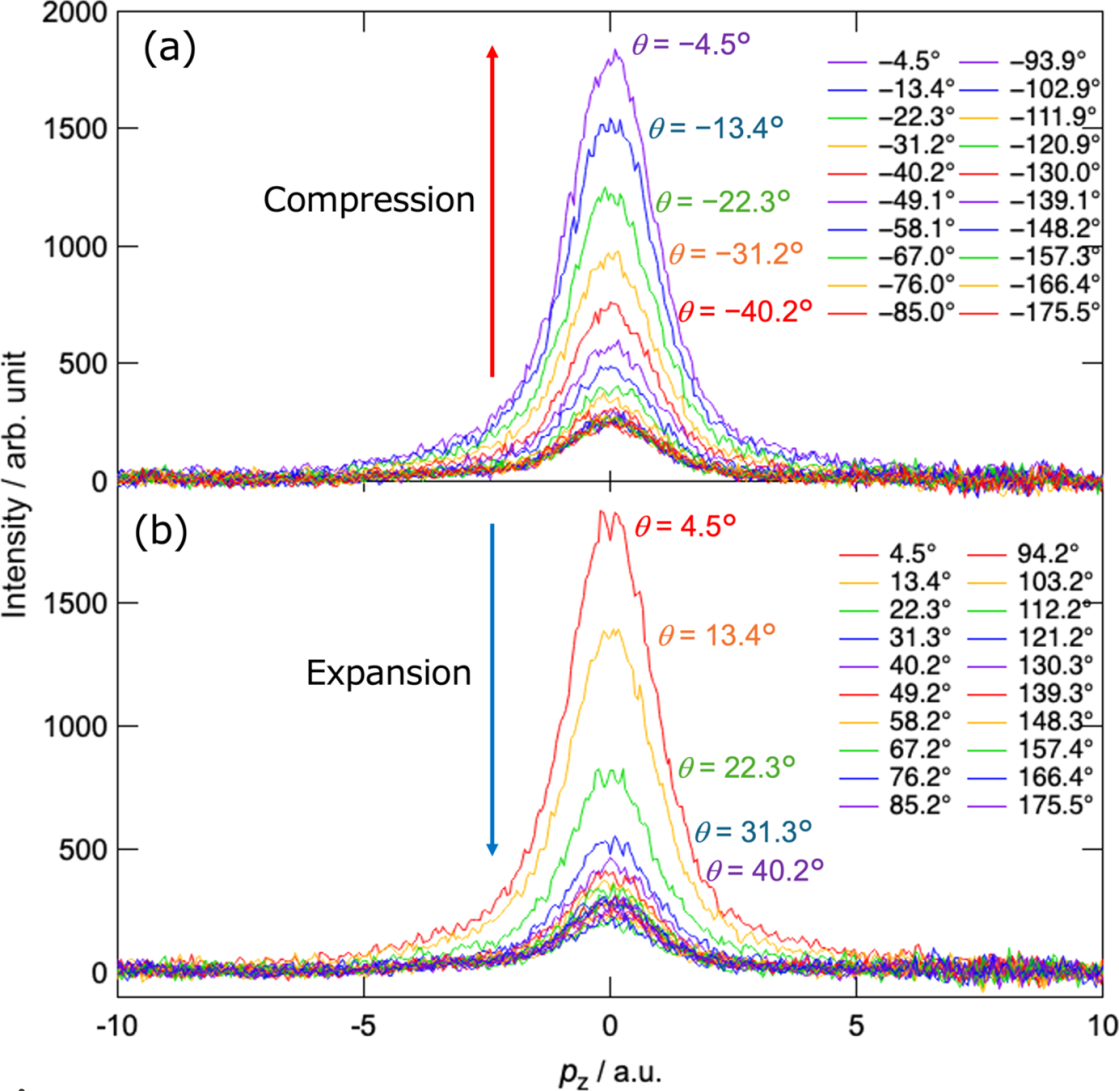
Time-resolved Compton-scattered X-ray spectra measured at the centre of the com­bus­tion chamber during piston motion. (*a*) Spectra divided into 20 intervals during the com­pres­sion process from BDC (crank angle −180°) to TDC (crank angle 0°) and (*b*) spectra divided into 20 intervals during the expansion process from TDC (crank angle 0°) to BDC (crank angle 180°).

**Figure 3:**
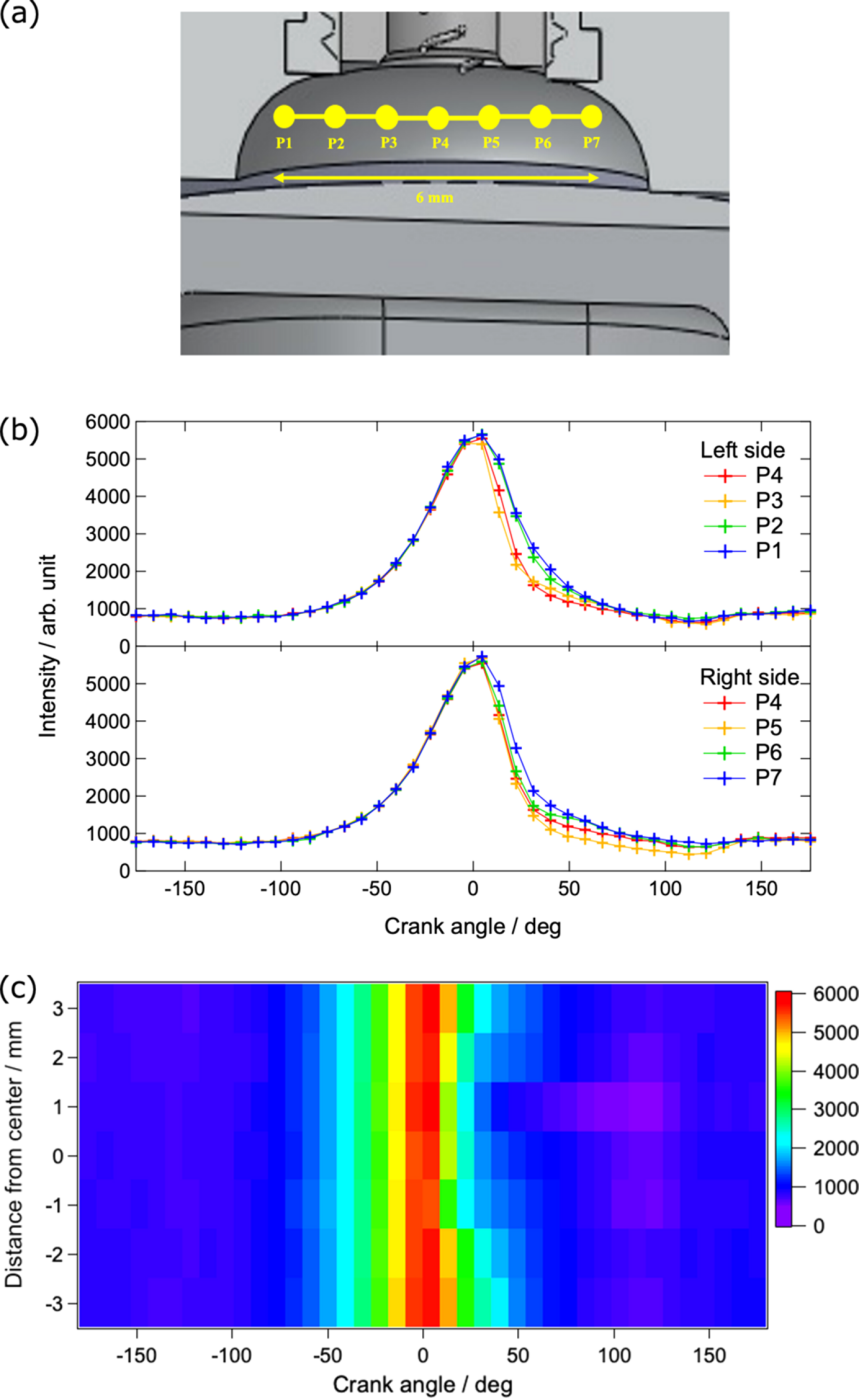
(*a*) Illustration of the interior of the com­bus­tion chamber, (*b*) crank-angle dependency of Compton-scattered X-ray intensities at positions P1–P7 and (*c*) corresponding two-dimensional map of the data shown in part (*b*). As illustrated in part (*a*), the measurement points in the com­bus­tion chamber, centred at P4 and spaced 1 mm apart, are indicated by yellow dots. On the vertical axis in part (*c*), P1 corresponds to −3 mm, P4 to 0 mm and P7 to 3 mm.

To analyze the temporal evolution of the Compton-scattered X-ray spectra, the time from BDC to TDC and that from TDC to BDC were each divided into 20 segments, resulting in a total of 40 segments per cycle. The X-ray signals within each segment were accumulated, and by repeating this process for multiple cycles, the Compton-scattered X-ray spectra at piston positions (crank angles) were obtained. Fig. 2 ▸ shows the crank-angle-dependent X-ray spectra at the centre of the com­bus­tion chamber [P4 in Fig. 3 ▸(*a*)] during com­pres­sion (crank angles θ = −180 to 0°) and expansion (crank angles θ = 0–180°), where θ values of −175.5 and 4.5° correspond to the states at the onset of com­pres­sion and expansion, respectively. Here, background X-rays were subtracted, as shown in the supporting information (Section S1) and the X-ray energy scale was converted to the electron momentum scale. The change in the spectra shows a gradual increase in intensity during com­pres­sion and an abrupt decrease during expansion.

The crank-angle dependence of the integrated intensity of the Compton-scattered X-ray spectra at positions P1 to P7 in the com­bus­tion chamber, as shown in Fig. 3 ▸(*a*), is presented in Fig. 3 ▸(*b*). P4 represents the centre of the com­bus­tion chamber, while P1–P3 are located on the left side and P5–P7 on the right side at 1 mm intervals. For comparison, the intensities at positions other than P4 were scaled relative to the onset of intensity increase at P4, assuming that pre-com­bus­tion intensity changes are independent of position. During the com­pres­sion process, the intensity begins to increase around a crank angle of −100°, when the exhaust port closes, and continues to rise until the piston reaches TDC (crank angle 0°). After reaching TDC, the intensity starts to decrease and does so at a faster rate than during the com­pres­sion process due to com­bus­tion. Around a crank angle of 120°, the intake port opens and an increase in intensity associated with gas exchange is observed. Since the intensity of Compton-scattered X-rays reflects the electron density over the probing volume, its variation corresponds to the change of gas density associated with piston motion and com­bus­tion. Fig. 3 ▸(*c*) shows a two-dimensional map of the crank-angle dependency of the integrated intensity at P1–P7 before and after com­bus­tion, with intensity represented on a colour scale. The rate of intensity decrease is slower at positions farther from the centre of the com­bus­tion chamber. The rapid decrease in intensity due to com­bus­tion begins around a crank angle of 13.5° at positions P3–P6 located near the centre of the com­bus­tion chamber, whereas at positions farther from the centre the intensity decrease occurs slightly later, around a crank angle of 22.5°. In addition, the rate of intensity decrease becomes slower toward the outer regions. This behaviour is likely caused by a reduction in flame propagation speed due to a decrease in the velocity of turbulence near the walls, as well as a decrease in the mixture temperature resulting from heat exchange with the walls, leading to delayed com­bus­tion or the presence of unburned gas. Therefore, by time-resolved Compton-scattering measurements of the gas inside the com­bus­tion chamber of an operating engine, we successfully captured intensity changes corresponding to density variations associated with piston motion and gas com­bus­tion.

**Figure 4:**
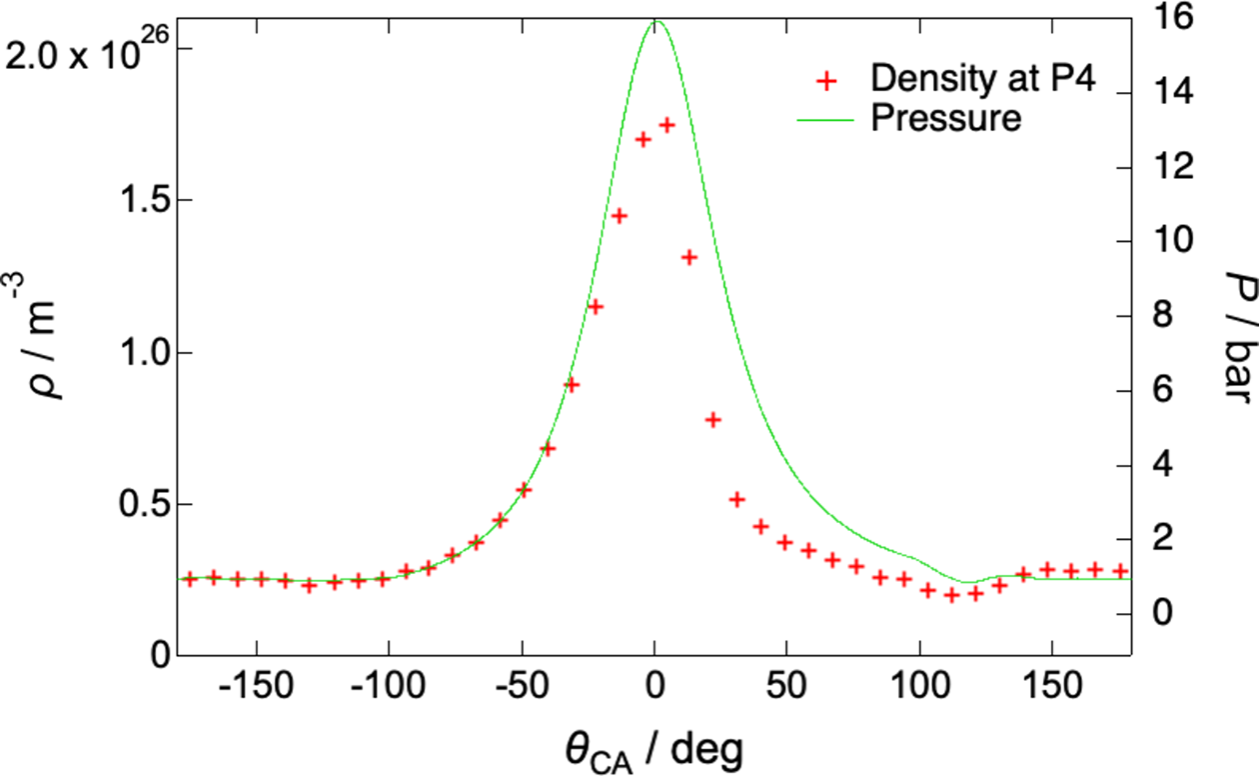
Comparison between the crank-angle dependency of molecular density at P4 and pressure. The red markers and green curve represent the molecular density and pressure, respectively. The pressure values shown are averages over 110 cycles at each crank angle, obtained while operating the model engine at a rotational speed of 8000 rpm. The molecular density is derived from the Compton-scattering X-ray intensity.

Pressure measurements in the com­bus­tion chamber were also conducted using the same type of model engine. This engine is equipped with a Kistler pressure sensor (type 6081A) to measure the pressure inside the com­bus­tion chamber. Fig. 4 ▸ shows a comparison between the crank-angle dependency of molecular density at P4 and pressure. Assuming that the molecular density is proportional to the Compton-scattering X-ray intensity, it is given by
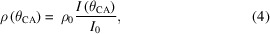
where  θ_CA_ is the crank angle, ρ_0_ = 2.5 × 10^25^ m^−3^ is the molecular density of the stoichiometric air–fuel mixture at 300 K and 1 bar, *I*(θ_CA_) is the Compton-scattering X-ray in­tensity measured at a crank angle θ_CA_ and *I*_0_ is the Compton-scattering X-ray intensity at a crank angle θ_CA_ of −175.5°. The pressure values shown are averages over 110 cycles at each crank angle, obtained while operating the model engine at a rotational speed of 8000 rpm. The pressure scale on the vertical axis is adjusted so that the rise in pressure coincides with the increase in the molecular density within the crank-angle range of −180 to −40°. The pressure increase during com­pres­sion was more pronounced than the increase in the molecular density and the subsequent pressure drop occurred more gradually than the decrease in the density. This difference is attributed to the temperature increase during com­pres­sion and com­bus­tion.

The gas temperature inside the com­bus­tion chamber can also be estimated using the Compton-scattered X-ray intensity and pressure. Assuming that the ideal gas equation of state holds, the temperature is proportional to the pressure divided by the X-ray intensity, as expressed by the following equation,
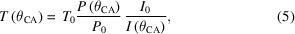
where *T*_0_ = 300 K is the temperature of the premixed air–fuel mixture before com­pres­sion, *P*(θ_CA_) is the pressure measured at a crank angle θ_CA_ and *P*_0_ is the pressure at a crank angle θ_CA_ of −175.5°. Since the pressure and intensity values are known, the temperatures at other crank angles can be roughly estimated. At a crank angle of approximately −100°, the exhaust port closes and the gas is subsequently compressed, leading to a rise in temperature. Near TDC (crank angle 0°), the gas temperature reaches approximately 700 K, which is consistent with the temperature increase expected from adiabatic com­pres­sion. In this engine, com­bus­tion is initiated by premixed com­pres­sion ignition of methanol. The auto-ignition temperature of methanol is 737 K (National Fire Protection Association, 2010 ▸), which is expected to be exceeded near the glow plug when the piston reaches TDC. Combustion is completed at a crank angle of 31.3° and the temperature reaches a maximum of 1100 K. Around a crank angle of 120°, the intake port opens and the com­bus­tion gas is gradually replaced by a room-temperature air–fuel mixture, resulting in a decrease in temperature to 300 K. These results show the capability of Compton scattering for estimating the temperature of in-cylin­der gases, together with pressure measurements.

It should be noted that the peak temperatures estimated in this study are lower than the temperature of static flames of propane around 1500 K (Sakurai *et al.*, 2016 ▸) and the adiabatic flame temperature of 2200 K for methanol (Haynes, 2015 ▸). This underestimation is most probably due to spatial and temporal averaging of the dynamically inhomogeneous gas temperature with the present experimental resolutions.

Finally, the temperature estimation in this work is based on the X-ray intensity analysis. An advanced analysis, such as the line-shape fitting, will improve the accuracy. A more detailed temperature evaluation based on model calculations is being conducted (Yamase *et al.*, 2026 ▸).

In conclusion, this article reports the first experiment of time-resolved Compton-scattering imaging with synchrotron-based high-energy X-rays on an operating internal com­bus­tion engine. The observed crank-angle dependency of Compton-scattered X-ray intensity allowed us to successfully capture the density changes of the gas in the com­bus­tion chamber associated with piston motion and com­bus­tion. Furthermore, by combining pressure measurements, the gas temperature in the com­bus­tion chamber of the operating engine was estimated successfully. This study demonstrates the capability of time-resolved Compton-scattering imaging as a unique technique to observe the dynamic behaviours of fuel and com­bus­tion gases in an internal com­bus­tion engine.

## Supplementary Material

Background subtraction from energy spectrum, conversion from Compton-scattered X-ray energy to electron momentum and ideal density as a function of crank angle. DOI: 10.1107/S1600577526002031/lin5001sup1.pdf
