# Supplementary material for: Imaging gas in a com­bus­tion engine with high-energy X-ray Compton scattering
Source: J Synchrotron Radiat. 2026 Apr 2;33(Pt 3):788–93. doi: 10.1107/S1600577526002031 (PMC13148619; doi:10.1107/S1600577526002031)
Supplement: Supplementary file 1 [file s-33-00788-sup1.pdf]

JOURNAL OF SYNCHROTRON RADIATION

Volume 33 (2026)

Supporting information for article:

Imaging gas in a combustion engine with high-energy X-ray Compton scattering

Yuki Mizuno, Hiroaki Suzuki, Naruki Tsuji, Takuyo Oguchi, Go Matsubara, Hiroyuki Yamase and Yoshiharu Sakurai

S1. Background subtraction from energy spectrum

To perform background measurements, it would be necessary to evacuate the combustion chamber of the engine, which is extremely difficult; therefore, in this study, the fitted results were used as the background. Figure S1 shows the fitting of the energy spectrum and the method used for background subtraction. The red curve is the energy spectrum obtained from Compton-scattered X-rays within the engine combustion chamber. The central peak around 93 keV is attributed to Compton-scattered X-rays from the gas in the combustion chamber. The other peaks are background signals, originating from lead fluorescence X-rays and Compton-scattered X-rays at different scattering angles. The energy spectrum was fitted using four Voigt functions. The fitted peak corresponding to the gas Compton peak around 93 keV was then removed, and the sum of the remaining three peaks was taken as the background. The difference between the original spectrum and the background can be converted into an electron momentum distribution, referred to as the Compton profile.

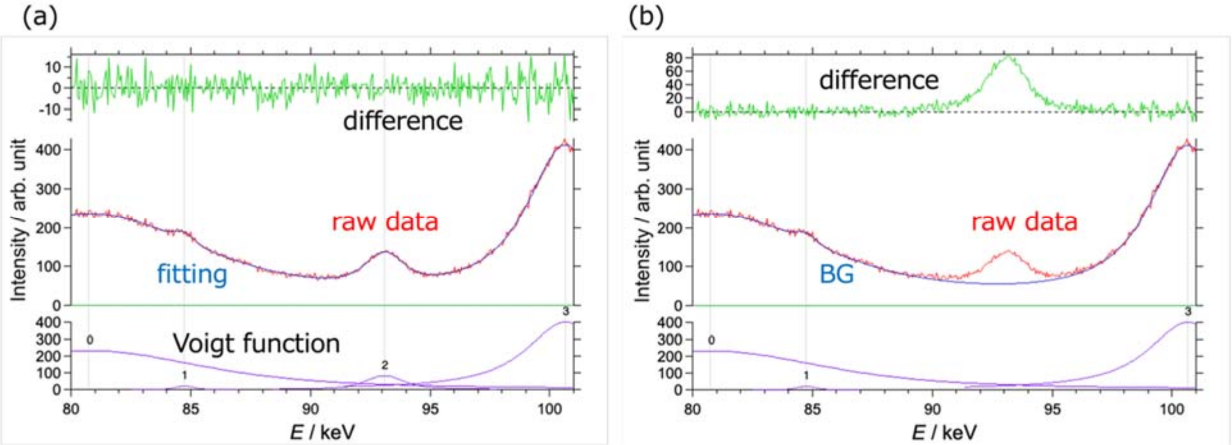

Figure S1 (*a*) Raw data and the fitting result by four Voigt functions. The red curve represents the raw energy spectrum obtained from Compton-scatterin X-rays within the engine combustion chamber. The blue curve shows the fitting result using four Voigt functions. The green curve represents the difference between the raw data and the fitting result. The purple curves correspond to the Voigt functions used in the fitting. (*b*) Background subtraction from energy spectrum. The blue curve represents the background obtained as the sum of the three Voigt profiles shown by the purple curves. The green curve shows the difference between the raw data and the background, which can be converted into an electron momentum distribution known as the Compton profile.

S2. Conversion from Compton-scattered X-ray energy to electron momentum

Conversion from the energy of Compton-scattered X-rays (ω_2) in keV to the electron momentum (p_z) in atomic units is provided by the following equation,

$$p_z = mc\frac{\omega_2 - \omega_1 + \frac{\omega_1\omega_2(1-\cos\theta)}{mc^2}}{\sqrt{\omega_1^2 + \omega_2^2 - 2\omega_1\omega_2\cos\theta}} \qquad \text{(S1)}$$

where ω_1 is the incident X-ray energy in keV, θ the scattering angle, m the rest mass of electron, and c the velocity of light.

S3. Ideal density as a function of crank angle

For comparison with the average density, Figure S2 shows the crank-angle dependence of the molecular density at the center of the combustion chamber and the reciprocal of the gas volume. The former is proportional to the local gas density, while the latter is proportional to the average gas density. The reciprocal of the volume is plotted over the crank-angle range from −100° to 100°, during which the exhaust port is closed, and the vertical axis is scaled to align with the rising trend of the molecular density at P4. After a crank angle of −13.5°, the molecular density decreases more rapidly than the reciprocal of the volume, indicating a local reduction in gas density associated with combustion.

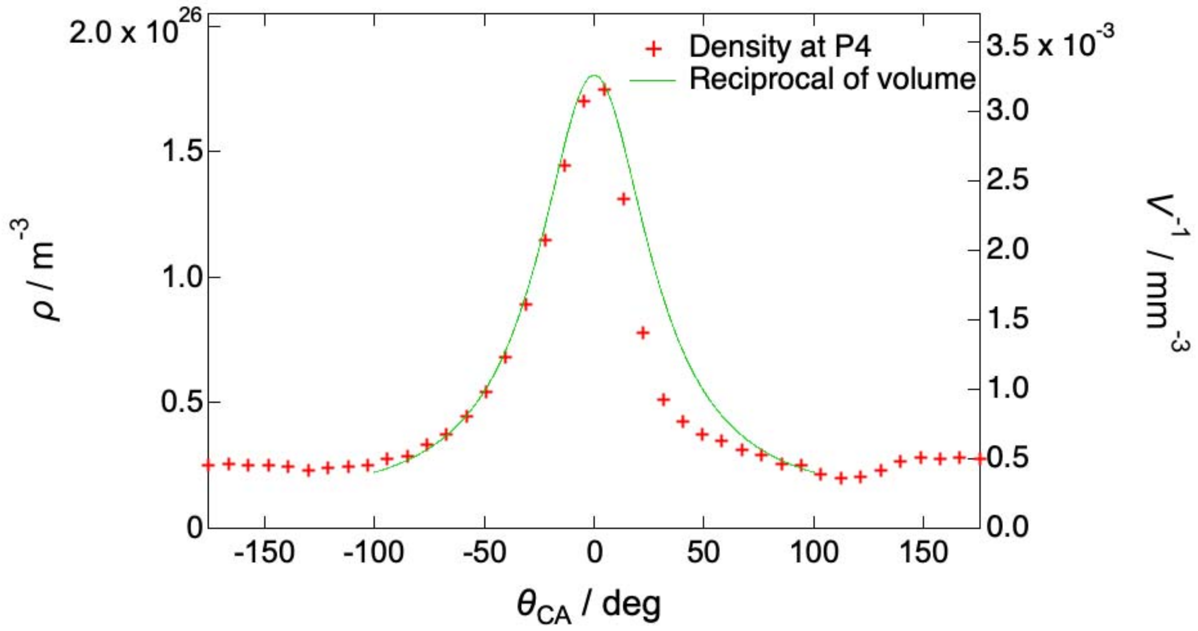

Figure S2 Comparison between the crank angle dependency of the molecular density at P4, and the reciprocal of the gas volume in the engine cylinder.
